# Establishing an assay to evaluate d‐amino acid oxidase enzyme kinetics and inhibition using WST‐8 redox dye

**DOI:** 10.1002/2211-5463.70217

**Published:** 2026-02-24

**Authors:** Kahoko Miyake, Yuki Enoki, Yuka Nakazawa, Kazuaki Taguchi, Kazuaki Matsumoto

**Affiliations:** ^1^ Division of Pharmacodynamics, Faculty of Pharmacy Keio University Tokyo Japan

**Keywords:** d‐amino acid, d‐amino acid oxidase, redox dye, uremic toxins, WST‐8

## Abstract

d‐Amino acid oxidase (DAO) inhibitors are novel candidate therapeutic agents for neurodegenerative diseases. In this study, a novel inhibition assay using WST‐8 as a detection reagent was developed and its effectiveness for evaluating redox‐based enzyme kinetics was tested. Furthermore, using this assay, we also investigated the inhibitory activities of uremic toxins and endogenous amino acid metabolites. Briefly, a mixture of DAO, WST‐8, and d‐amino acids was allowed to react in phosphate buffer and DAO activity was detected as an increase in absorbance when both d‐serine and DAO were present. However, as reducing compounds could potentially cause false‐positive results, we confirmed that the detection of DAO activity in this system was mediated by the redox reaction of WST‐8. Critically, we verified that this assay could be used to evaluate the inhibitory activity of known DAO inhibitors, including uremic toxins which showed weak DAO inhibition. This study establishes an alternative assay system that can be utilized for assessing DAO‐inhibitory activity and other enzymatic reactions mediated by redox reactions.

## Graphical abstract

This study investigated a novel WST‐8‐based assay for evaluating d‐Amino acid oxidase (DAO) inhibitors. We confirmed its effectiveness using known inhibitors and found that uremic toxins possess relatively weak inhibitory activity compared to existing drugs. Thus, this method provides a reliable alternative for assessing DAO inhibition and related enzymatic redox reactions.

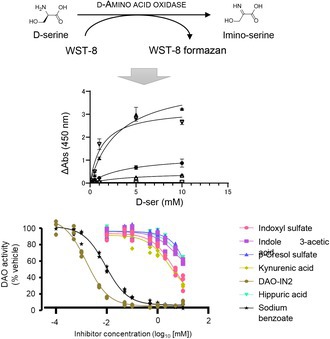

**Abbreviations**
CKD: chronic kidney disease
DAO: d‐amino acid oxidase
FAD: flavin adenine dinucleotide
HRP: horseradish peroxidase
NMDAR: N‐methyl‐*d*‐aspartate receptor

d‐Amino acid oxidase (DAO), a flavoprotein belonging to the dehydrogenase/oxidase family, uses flavin adenine dinucleotide (FAD) as a cofactor [1]. DAO catalyzes the oxidative deamination of d‐amino acids, producing the corresponding α‐keto acids, ammonia, and hydrogen peroxide. Recent studies have elucidated the physiological roles of d‐amino acids in mammals. Among various d‐amino acids, d‐serine (d‐Ser) acts as a co‐agonist of N‐methyl‐*d*‐aspartate receptors (NMDARs) in the brain, modulating synaptic plasticity and neurotransmission. The dysfunction of these receptors has been implicated in various pathological conditions. For instance, excessive activation of NMDARs is associated with disorders such as epilepsy, Parkinson's disease, Alzheimer's disease, and amyotrophic lateral sclerosis, whereas their insufficient activation receptors is associated with neuropsychiatric disorders, such as schizophrenia [2, 3, 4, 5, 6]. DAO, which regulates the levels of the co‐agonist d‐serine and thereby modulates NMDAR activation, is closely involved in the pathophysiology of these disorders. Consequently, small molecules that modulate DAO activity have attracted attention as potential therapeutic agents for NMDAR‐related diseases [7]. Several DAO inhibitors have been developed for clinical applications. For instance, benzoate is a DAO inhibitor that has been studied extensively as a therapeutic agent for various neuropsychiatric disorders, including schizophrenia [8]. Thus, DAO inhibitors are promising candidates for the development of novel pharmacological interventions.

Various methodologies have been reported for assessing DAO activity, including monitoring oxygen consumption, employing electron‐acceptor‐based reduction assays, and quantifying the levels of α‐keto acids, hydrogen peroxide, and ammonium ions [9]. Among these methods, the reduction dye assay (e.g., 2,6‐dichlorophenol‐indophenol, methylene green, and thionine) is the most convenient and cost‐effective [10]. However, these methods have been criticized for their limited sensitivity in detecting DAO activity, despite their simplicity and low cost [9]. The Amplex Red® assay, a highly sensitive method, detects DAO activity in the range of 0.25–2.5 mU·mL^−1^ [11, 12]. In contrast, Brugger et al. reported that the reduction dye assay has a detection range of 500–5000 DAO mU·mL^−1^ [10], demonstrating a sensitivity difference of up to 2000‐fold between the two methods. However, the Amplex Red® method is more expensive than the reduction dye assay because it requires an expensive fluorescent probe, in addition to multiple reagents, such as horseradish peroxidase (HRP) and FAD, and a fluorescence plate reader (which is more costly than an absorbance plate reader). Furthermore, systems that detect ammonia or α‐keto acids require coupling reactions for detection, whereas the Amplex Red® method involves a coupling method with HRP, which is challenging to perform, unlike the reduction dye assay, which is relatively simple [9]. Notably, the enzyme activity in a Michaelis–Menten type enzymatic reaction is enhanced when the substrate concentration equals the enzyme *K*_m_ value [*K*_m_ = (S)] [13]. Therefore, it is necessary to standardize the assay using the *K*_m_ of DAO and the optimal concentration of d‐amino acids.

The WST‐8 reagent allows for the easy assessment of cell viability; the reagent is simply added to cultured cells and is absorbed at 450 nm when reduced [14, 15]. Notably, WST‐8 works at physiological pH, remains stable for extended periods, even when incubated at 37 °C, and demonstrates sufficient sensitivity, even in small cell volumes cultured in 96‐well plates. These advantages make it suitable for the detection of enzyme reactions in terms of temperature, pH, and sensitivity. This method is easy to apply because it can be performed in a one‐step process. Therefore, this study aimed to develop a simple assay for evaluating DAO‐inhibitory activity using WST‐8.

Uremic toxins are a group of metabolites that accumulate in the body during diseases associated with impaired kidney function, such as chronic kidney disease (CKD) [16]. Among the uremic toxins, some protein‐bound molecules are amino acid metabolites. For instance, indoxyl sulfate and indole acetic acid are metabolites of tryptophan, with sulfate and acetate groups bound to the indole backbone [17]. Additionally, p‐cresyl sulfate is a metabolite of tyrosine and phenylalanine, in which the hydroxyl group of cresol undergoes sulfate conjugation [17, 18]. The structures of these compounds are similar to those of known DAO inhibitors [19]. Consequently, it is plausible that amino acid derivatives possess inhibitory activity because DAO metabolizes amino acids. Based on these considerations, we hypothesized that uremic toxins derived from amino acids are endogenous compounds with DAO‐inhibitory activity.

Therefore, this study aimed to establish a simple and cost‐effective method using the WST‐8 redox dye for use as a screening system for DAO inhibitors and to explore the potential of WST‐8 as a detection reagent for redox enzyme reactions. Additionally, we evaluated the DAO‐inhibitory activity of uremic toxins and endogenous amino acid metabolites using the developed assay.

## Methods

### Materials

WST‐8 (Cell Counting Kit‐8) was purchased from Dojin Chemical Co. Ltd. (Kumamoto, Japan). Porcine kidney DAO (A5222) was purchased from Millipore (Billerica, MA, USA). d‐Alanine (d‐Ala), d‐Ser, d‐aspartic acid (d‐Asp), d‐glutamic acid (d‐Glu), d‐arginine (d‐Arg), D‐lysine (d‐Lys), d‐histidine (d‐His), d‐valine (d‐Val), d‐leucine (d‐Leu), d‐threonine (d‐Thr), d‐cysteine (d‐Cys), d‐methionine (d‐Met), d‐asparagine (d‐Asn), d‐phenylalanine (d‐Phe), d‐tyrosine (d‐Tyr), d‐tryptophan (d‐Trp), sodium benzoate (SB), p‐cresol sulfate, indoleacetic acid (IA), kynurenic acid (KA), and hippuric acid (HA) were purchased from Tokyo Kasei (Tokyo, Japan). d‐Isoleucine (d‐Ile), d‐glutamine (d‐Gln), d‐proline (d‐Pro), l‐cysteine (l‐Cys), N‐acetyl‐*l*‐cysteine, sodium dihydrogen phosphate, disodium hydrogen phosphate, acetonitrile, methanol, ethanol, and ultrapure water were purchased from Fujifilm Wako Pure Chemical Industries (Osaka, Japan). Trifluoroacetic acid was purchased from NACALAI TESQUE, Inc. (Kyoto, Japan). Potassium indoxysulfate salt was purchased from Santa Cruz Biotechnology (Dallas, TX, USA). DAO‐IN‐2 was purchased from MedChemExpress (Monmouth Junction, NJ, USA).

### Evaluation of DAO activity using the redox dye WST‐8

DAO and d‐amino acids were dissolved in phosphate buffer (0.2 m, pH 8.0). d‐Tyr and d‐Trp were dissolved in 0.1 N NaOH and adjusted to approximately pH 8.0 using 0.1 N HCl. WST‐8 (final conc. 10–50%), DAO (final conc. 0.01–1.0 U·mL^−1^), and d‐amino acids (final conc. 2 mm) were added to a 96‐well plate at one‐tenth of the final volume, and baseline absorbance (450 nm) was measured. The plate was then incubated at 37 °C on a hot plate, and time‐dependent changes (0–120 min) in absorbance were recorded.

### Temperature and pH dependence of DAO activity

In the temperature‐dependent experiments, WST‐8 (final conc. 10%), DAO (final conc. 0.2 U·mL^−1^), and d‐Ser (final concentrations 2 mm) were added to a 96‐well plate at one‐tenth of the final volume, and baseline absorbance (450 nm) was measured. The plate was then incubated at 10–60 °C on a hot plate for 60 min, and absorbance was recorded.

In the pH‐dependent experiments, WST‐8 (final conc. 10%), DAO (final conc. 0.2 U·mL^−1^), and d‐Ser (final concentrations 2 mm) were added to a 96‐well plate at one‐tenth of the final volume, and baseline absorbance (450 nm) was measured. Residual activity at different pH values was assayed in a multicomponent buffer (15 mm Tris–HCl, 15 mm Na_2_CO_3_, 15 mm H_3_PO_4_, 100 mm KCl, and 1% (v/v) glycerol) described previously [20] (Fig. S1).

### Validation assay

The specificity of the reaction system was evaluated using l‐Ser as a negative control. In the validation assay, WST‐8 (final conc. 10%), DAO (final conc. 0.2 U·mL^−1^), and d‐Ser (final concentrations. 0.1–10 mm) were added to a 96‐well plate at one‐tenth of the final volume, and baseline absorbance (450 nm) was measured. The plate was then incubated at 37 °C on a hot plate for 60 min, and absorbance was recorded.

### Measurement of d‐ser concentrations using liquid chromatography–tandem mass spectrometry (LC–MS/MS)

The residual d‐Ser was quantified to confirm whether the observed reaction was due to d‐Ser degradation. Briefly, calibration standards for d‐Ser (0, 0.3125, 0.625, 1.25, 2.5, and 5 mm) were prepared by dissolving in water (LC–MS grade, Fujifilm Wako Pure Chemicals, Chuo‐ku, Osaka, Japan). WST‐8 (10%) and d‐Ser (2 mm) were added to the phosphate buffer and incubated at 37 °C for 60 min in the presence or absence of DAO (0.2 U·mL^−1^). The reaction solution was deproteinized using acetonitrile (1 : 9) and analyzed using a mass spectrometer (Shimadzu Co. Ltd., Kyoto, Japan) according to a previously reported method [21]. The LC–MS/MS conditions are described in detail in Table S1.

### Development of a DAO‐inhibitory activity assay

SB, IS, IA, and HA were dissolved in 0.2 m phosphate buffer. DAO‐IN‐2, IA, and KA were dissolved in 0.1 N NaOH and adjusted to approximately pH 8.0 with 0.1 N HCl. The final concentrations of SB and DAO‐IN2 were 0.00001–10 mm and those of uremic toxins were 0.01–10 mm. Briefly, 60 μL of phosphate buffer, 10 μL of WST‐8, 10 μL of DAO (final conc. 0.2 U·mL^−1^), 10 μL of inhibitor (dissolved in phosphate buffer), and 10 μL of d‐Ser (final conc.2 mm) were mixed in a 96‐well plate and the baseline absorbance (450 nm) was measured to evaluate DAO‐inhibitory activity. The plate was then incubated at 37 °C for 60 min, and the absorbance was measured again. The modes of inhibition were evaluated using reciprocal plots.

Dilution and pH alterations were examined to investigate methods of terminating the reaction. After a 60‐min reaction at 37 °C, the absorbance was measured. In dilution method (1), an equal volume of ultrapure water was added to the reaction mixture to stop the reaction. In the pH alteration method (2), 1–10 N hydrochloric acid (acidic condition) or (3) 1–5 N sodium hydroxide (alkaline condition) was added at one‐tenth the volume of the reaction mixture. After each reaction was terminated, the samples were incubated at room temperature for 60 min, the absorbance was measured, and the rate of change was evaluated.

### Enzyme kinetic parameters analysis

Kinetic constants were calculated using nonlinear least‐squares regression, fitting the data to the Michaelis–Menten equation. Additionally, the half maximal inhibitory concentration (IC_50_) was calculated using graphpad prism version 10 for Windows (GraphPad Software, Boston, MA, USA). The mode of inhibition was evaluated using a double reciprocal (Lineweaver–Burk) plot analysis.

## Results

### Determination of DAO activity using WST‐8

**Fig. 1:**
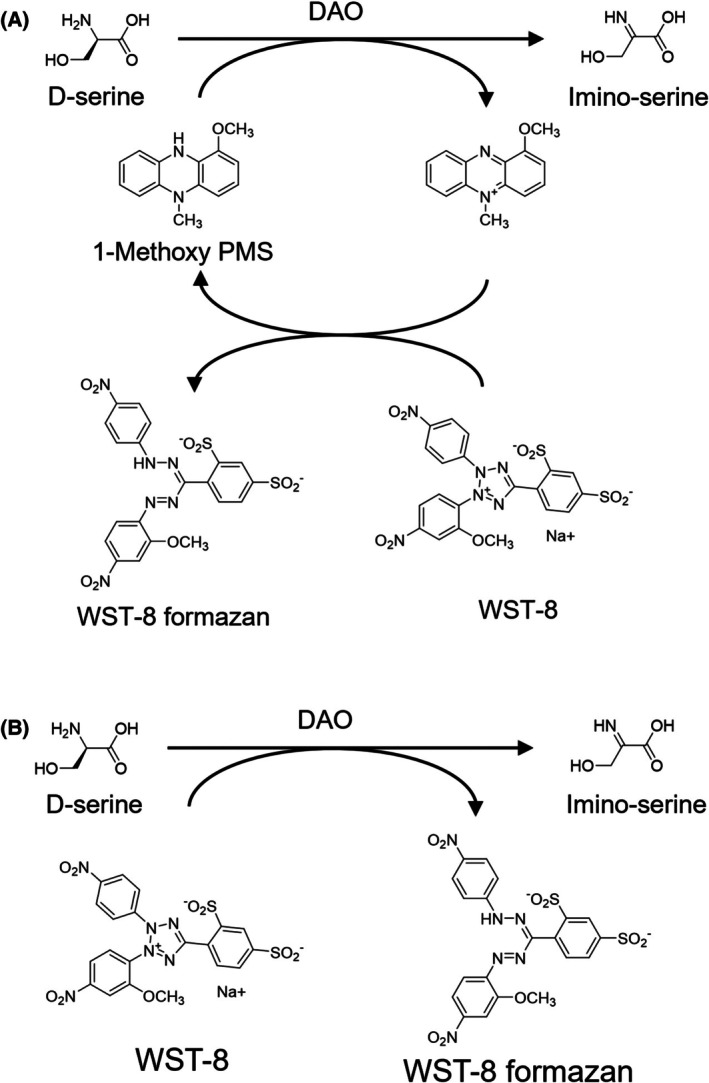
Proposed reaction scheme. d‐Amino acid oxidase (DAO) activity was detected by the indirect conversion of WST‐8 to WST‐8 formazan, either (A) via 1‐methoxy PMS or (B) directly through electron transfer.

**Fig. 2:**
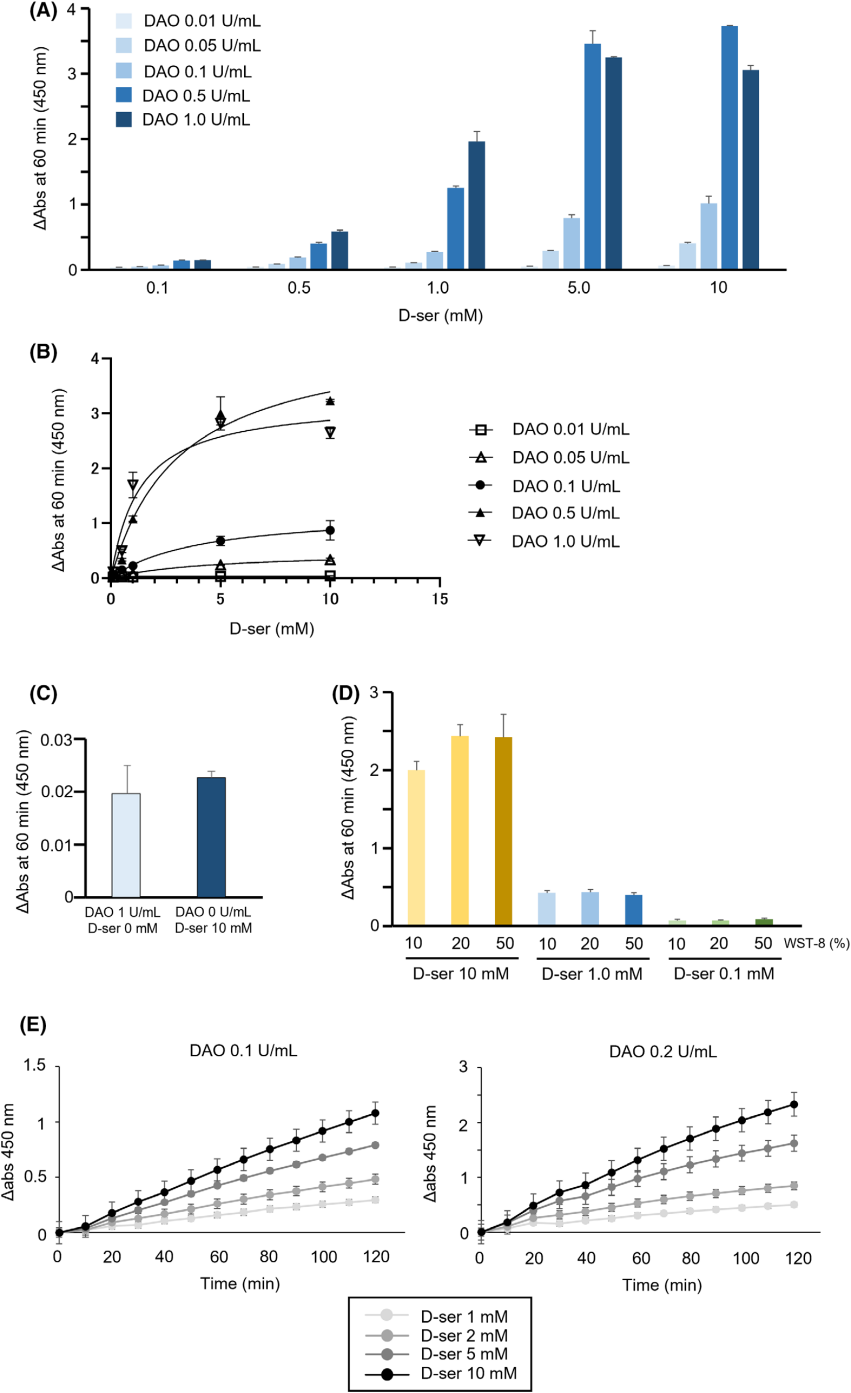
Evaluation of the utility of WST‐8 for DAO activity detection. (A) Dose dependency of d‐amino acid oxidase (DAO; 0.01–1.0 U·mL^−1^) and d‐serine (d‐Ser; 0.1–10 mm) at 37 °C for 60 min. (B) Kinetic analysis of DAO by a Michaelis–Menten type enzymatic reaction. (C) Change in absorbance in the absence of DAO or d‐serine. DAO was used at 1 U·mL^−1^ and d‐serine at 10 mm. (D) Investigation of the optimal concentration of WST‐8. WST‐8 concentrations were tested ranging from 10 to 50%. d‐Serine was tested at 0.1–10 mm and DAO at 0.2 U·mL^−1^. (E) Examination of the optimal reaction conditions (DAO units, d‐serine concentration, incubation time). d‐Serine was tested at concentrations of 1–10 mm, DAO at 0.1 or 0.2 U·mL^−1^, and measurements were taken every 10 min up to 120 min. Data are expressed as means ± SE. *n* = 3 per experiment.

The proposed reaction scheme is illustrated in Fig. 1. DAO metabolizes D‐Ser, and the electrons generated in this process reduce WST‐8 either indirectly via PMS‐1 or directly through a reduction reaction. First, various concentrations of d‐Ser and DAO were reacted in the presence of WST‐8. The absorbance of WST‐8 increased with increasing concentrations of d‐Ser and DAO (Fig. 2A). Based on these values, an analysis using the Michaelis–Menten equation showed that DAO activity remained relatively stable at 0.05–0.5 U·mL^−1^, with an average *K*_m_ value ranging from 3.151 (2.166 to 4.785) to 4.527 (2.949 to 7.463) (Table 1 and Fig. 2B). In addition, in the absence of d‐Ser or DAO, almost no increase in absorbance was observed (Fig. 2C). Subsequently, we examined the effects of various WST‐8 concentrations. A final concentration of 10% WST‐8 was sufficient for DAO detection (Fig. 2D). Furthermore, an absorbance value of < 3.0, which is generally considered the upper limit for spectrophotometric assays, fell within the range recommended by the supplier of the WST‐8 reagent. Absorbance increased linearly up to 120 min at 0.1 and 0.2 U·mL^−1^ DAO at any D‐ser concentration (Fig. 2E).

**Table 1:** Apparent kinetic parameters. Data are expressed as the most fitted value (95% confidence interval).

DAO U·mL^−1^	*V*_max_ (abs. 450 nm)	*K*_m_ (mm)
0.01	0.0330 (0.02659–0.04201)	0.1257 (0.01646–0.5763)
0.05	0.4788 (0.4052–0.6010)	4.527 (2.949–7.463)
0.1	1.226 (0.9871–1.694)	4.068 (2.278–8.292)
0.5	4.467 (3.901–5.260)	3.151 (2.166–4.785)
1.0	3.250 (2.826–3.759)	1.309 (0.8250–2.117)

**Fig. 3:**
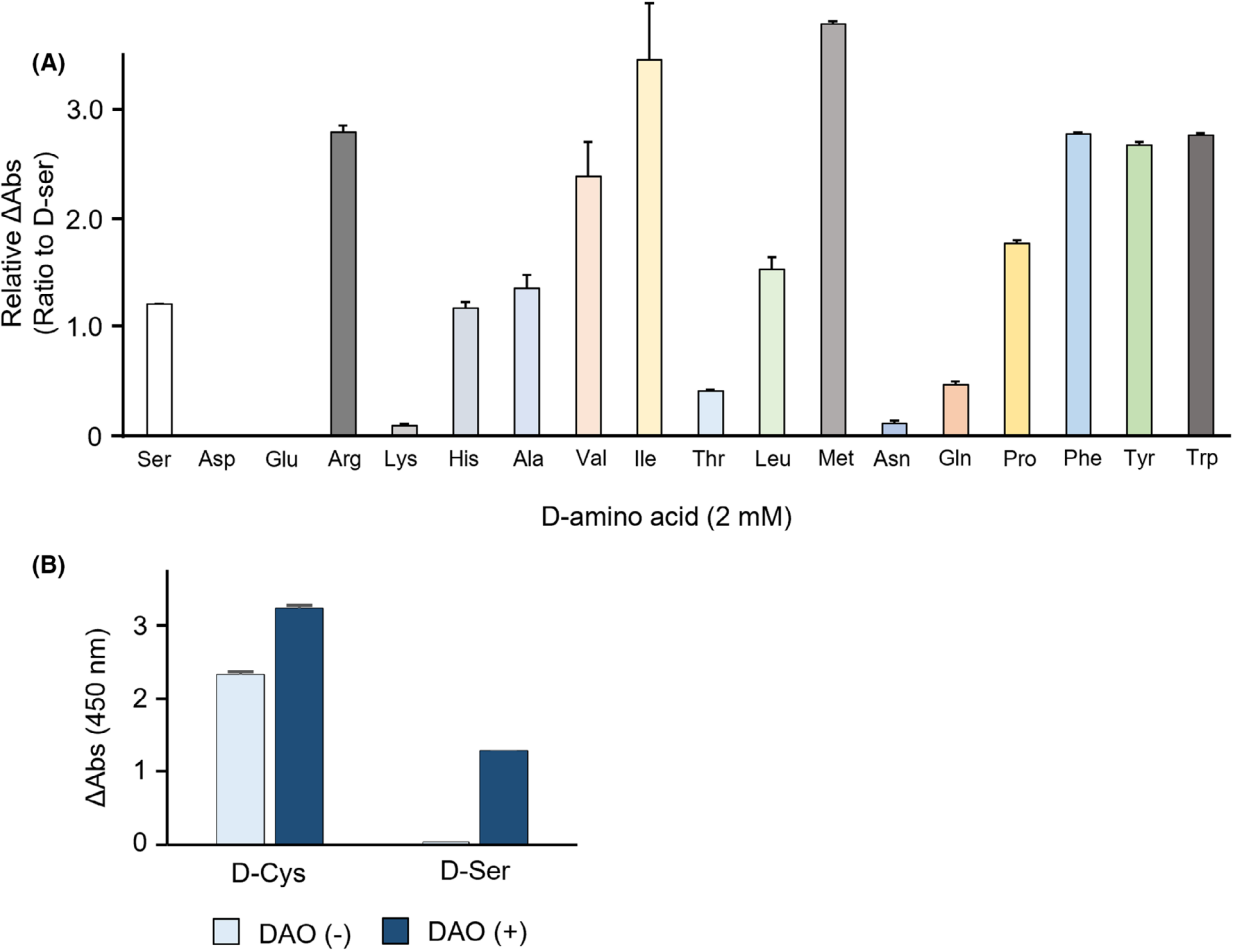
Reactivity of assay with various d‐amino acids. (A) Reactivity of the assay with various d‐amino acids (2 mm). d‐Amino acid oxidase (DAO) was used at 0.2 U·mL^−1^, with reaction conditions set at 37 °C for 60 min. (B) Reactivity of d‐cysteine (d‐Cys) with or without DAO. Each d‐amino acid was used at 2 mm, DAO at 0.2 U·mL^−1^, and the reaction was carried out at 37 °C for 60 min. Data are expressed as means ± SE. *n* = 3 per experiment.

We investigated the amount of residual d‐Ser in the reaction mixture after 1 h at 0.2 U·mL^−1^ DAO. Notably, this reaction resulted in the degradation of all d‐amino acids, except for d‐Asp and d‐Glu (Fig. 3A), indicating that the reaction detected the degradation of d‐amino acids by DAO. However, an increase in absorbance was observed for d‐Cys even in the absence of DAO (Fig. 3B). Therefore, l‐Cys and a known reductant, N‐acetylcysteine, were examined under DAO‐free conditions to investigate the potential of the thiol group in Cys as a reducing agent. An increase in WST‐8 absorbance was observed, indicating that the reaction with d‐Cys was DAO‐independent (Fig. S4).

### Evaluation of the DAO‐inhibitory activity assay

**Fig. 4:**
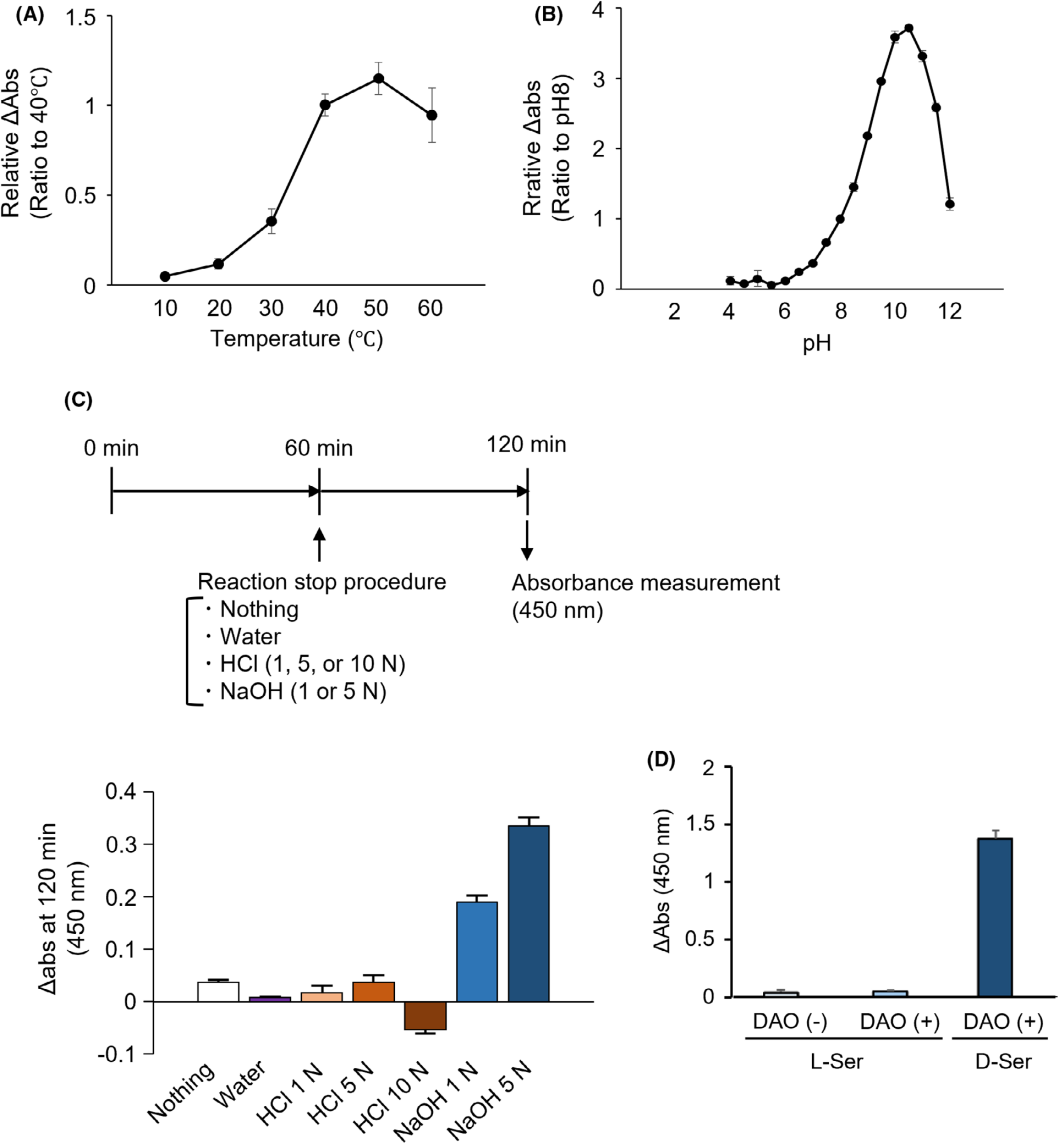
Effects of temperature and pH on DAO enzymatic activity. d‐Amino acid oxidase (DAO) was fixed at 0.2 U·mL^−1^ and d‐serine at 2 mm, and the effects of (A) temperature and (B) pH on enzyme activity were assessed. (C) The effect of pH changes on the reaction stop conditions after the 120 min reaction was evaluated. (D) Specificity evaluation using l‐Ser or d‐Ser with or without DAO. Data are expressed as means ± SE. *n* = 3 per experiment.

We examined the temperature and pH dependence of DAO activity during the assay development process to assess whether the WST‐8‐based assay system was suitable for evaluating compounds with DAO‐inhibitory activity (Fig. 4). Enzyme activity increased with temperature, peaking at 50 °C. However, minimal differences in activity were observed between 40 °C and 60 °C (Fig. 4A). Therefore, the inhibition assay temperature was set to 37 °C, given that the ultimate goal is to evaluate inhibitory activity under physiological conditions. In addition, no reaction was observed at 4 °C (Fig. S5), which indicated that lowering the reaction temperature was sufficient to effectively terminate the enzymatic reaction. Subsequent investigation of the pH dependence revealed that the absorbance increased with increasing pH, reaching its maximum at pH 10 (Fig. 4B). We selected pH 8 for our inhibition assay to maintain consistency with most previously reported DAO inhibition assays. In contrast, the absorbance decreased at pH values of > 10, and a distinct color change was observed at pH ≥ 11 (Fig. S1). Therefore, we investigated whether dilution or the use of pH‐modifying agents could serve as methods to stop the reaction. Dilution by more than two‐fold and mild acidification were acceptable (Fig. 4C). However, WST‐8 formazan reagent was not stable under strongly alkaline conditions (Fig. 4C) [22]. Additionally, making the solution strongly alkaline was deemed unsuitable as a method to stop the reaction because it caused a significant change in absorbance. Thus, considering the simplicity of the assay, no special procedure was necessary to terminate the reaction. Simply removing the plate from the 37 °C hot plate to allow the temperature to drop was sufficient for practical use.

### Validation of the assay and evaluation of the DAO‐inhibitory activity of uremic toxins

Specificity was evaluated by comparing results using d‐Ser and l‐Ser. No reaction was observed for l‐Ser, whereas an increase in absorbance was observed only in the presence of d‐Ser (Fig. 4D). The limit of detection (sensitivity) was initially estimated to be 0.1 mm d‐Ser. However, validation results showed that the accuracy at 0.1 mm d‐Ser was 170%, indicating a substantial deviation (Table 2, Fig. S6). Accordingly, the practical lower limit (sensitivity) was determined to be 0.2 mm. In addition, accuracy, precision, and reproducibility were all within 25%, even at the lower limit.

**Table 2:** Analytical validation. SD, Standard deviation.

	Target conc. (mm)	Average conc. ± SD (mm)	Precision (CV%)	Accuracy (%)
d‐Ser	0.1	0.17 ± 0.02	11.99	170.0
	0.2	0.25 ± 0.01	5.56	122.8
	0.5	0.480 ± 0.02	3.80	96.9
	1	0.90 ± 0.04	4.61	90.2
	5	5.24 ± 0.81	15.49	104.8
	10	9.94 ± 1.87	18.82	99.4

**Fig. 5:**
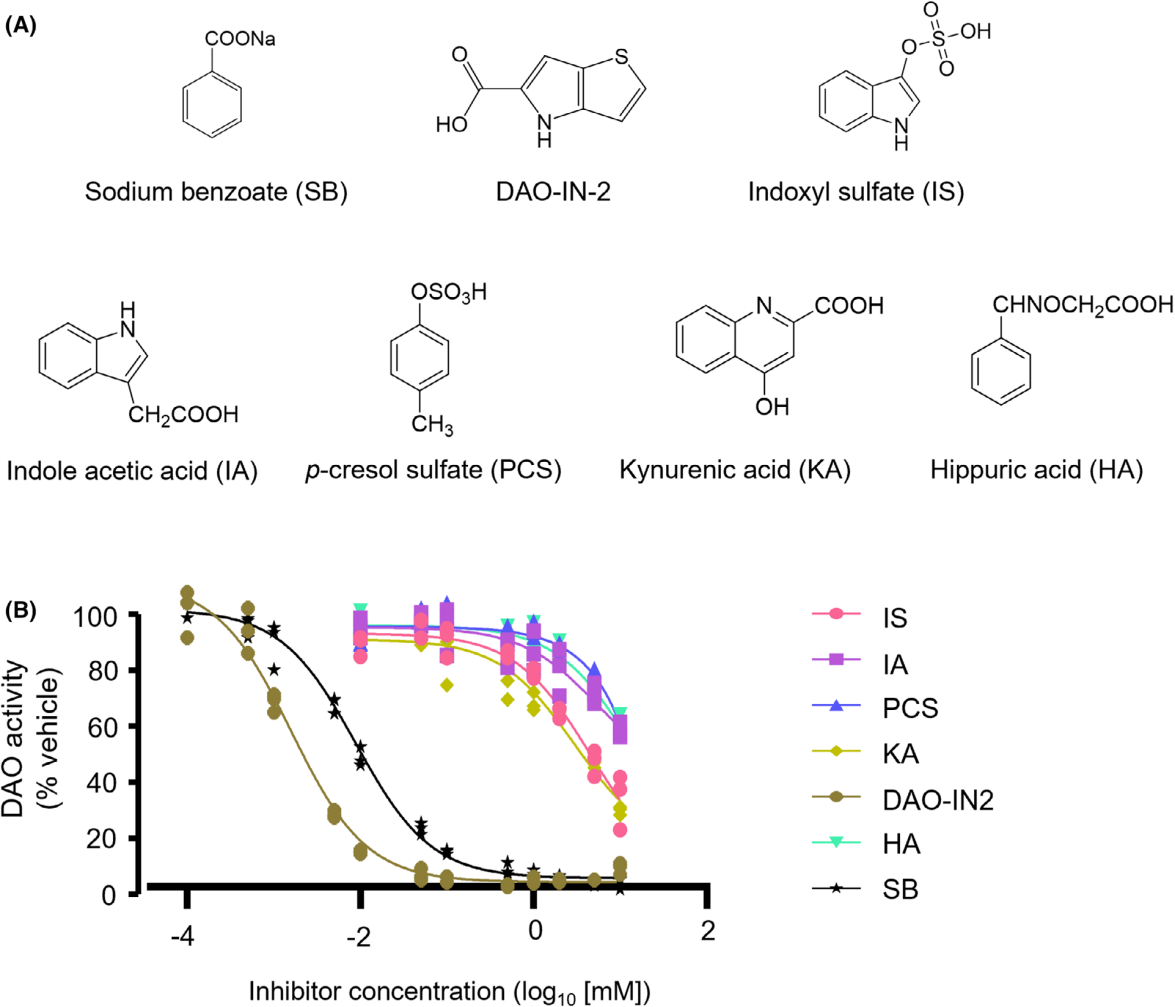
Validation of the inhibitory activity assay using known DAO inhibitors and assessment of the inhibitory activity of uremic toxins. (A) Chemical structures of the tested compounds (sodium benzoate [SB], DAO‐IN‐2, indoxyl sulfate [IS], indoxyl acetic acid [IA], *p*‐cresol sulfate [PCS], kynurenic acid [KA], and hippuric acid [HA]). (B) Evaluation of the concentration dependence of inhibitory activity. All experiments were conducted in triplicate. DAO, d‐amino acid oxidase.

Using this assay, we evaluated the inhibitory activity of uremic toxins, which are amino acid‐derived metabolites, using SB and DAO‐IN‐2, known DAO inhibitors, as positive controls (Fig. 5A). First, a decrease in absorbance was observed with the addition of SB and DAO‐IN‐2, confirming that this assay system was capable of evaluating the DAO‐inhibitory activity (Fig. 5B). Among the uremic toxins, IS [IC_50_ (mm): 4.52 (2.775–8.094)], IA [IC_50_ (mm): 5.36 (1.814–38.03)], and KA [IC_50_ (mm): 2.96 (1.390–7.277)], which possess indole rings, exhibited weaker inhibitory activity than that of SB [IC_50_ (mm): 0.0016 (0.00129–0.00197)] and DAO‐IN‐2 [IC_50_ (mm): 0.0089 (0.00756–0.01046)] (Fig. 5B and Table 3). Finally, the mode of inhibition was evaluated using a reciprocal plot, which confirmed that the inhibitory activity of DAO was primarily competitive (Fig. S7).

**Table 3:** Apparent kinetic parameters of DAO inhibitors and uremic toxins.

Inhibitors	IC_50_ (mm)	Ref.
Indoxyl sulfate	4.52 (2.775–8.094)	‐
Indole acetic acid	5.36 (1.814–38.03)	‐
*p*‐Cresol sulfate	> 100	‐
Kynurenic acid	2.96 (1.390–7.277)	‐
Hippuric acid	13.15 (5.205–155.8)	‐
DAO‐IN‐2	0.0016 (0.00129–0.00197)	245 ± 170 nm ^a^
Sodium benzoate	0.0089 (0.00756–0.01046)	10 μm ^b^

^a^ Human DAO [Spsrey T. *Bioorg Med Chem Lett* 2008]

^b^ Human DAO [Toguchi S. *Chem Pharm Bull*. 2016].

## Discussion

This study established a simple DAO‐inhibitory activity evaluation system using the one‐step reagent WST‐8, which is commonly used in live cell assays. Furthermore, using this system, we demonstrated that uremic toxins, such as IS and IA, which are metabolites of amino acids *in vivo*, exhibit very weak DAO‐inhibitory activity.

DAO metabolizes d‐amino acids to produce imino acids while reducing FAD to FADH_2_. This reduction reaction has been used to detect DAO activity using redox dyes [10]. Additionally, a previous study detected DAO activity based on a reduction‐induced decrease in the absorbance of 2,6‐dichlorophenol‐indophenol (520 nm), methylene green (655 nm), and thionine (600 nm). In contrast, in the present study, we used the WST‐8 reagent, which exhibited an increase in absorbance at 450 nm upon reduction. WST‐8 is a commonly used cell viability assay based on the reduction of the electron mediator 1‐methoxy PMS by intracellular NADH, which subsequently reduces WST‐8 to WST‐8 formazan, resulting in increased absorbance at 450 nm. Accordingly, in the presence of DAO and d‐Ser, an increase in absorbance at 450 nm was observed in a concentration‐dependent manner. Furthermore, similar results were obtained when other d‐amino acids were tested, except d‐Asp and d‐Glu, which have been reported to be less susceptible to the action of DAO [23]. Given that the reaction rate varies depending on the amino acid [23], this result was expected. In addition, an increase in absorbance was observed with d‐Cys, independent of the DAO activity, which was presumed to be a false‐positive result due to the chemical reduction of WST‐8. The manufacturer reported that WST‐8 could also be reduced by common reducing agents, such as ascorbic acid. Accordingly, the remaining d‐Ser content was 28% (approximately 0.56 mm), indicating that the increase in absorbance was due to d‐Ser degradation. Thus, combined with the results shown in Fig. 2A, these findings indicated that the reaction decreased because of a reduction in the substrate concentration caused by d‐Ser degradation (Fig. S3). Taken together, these findings indicate that the present assay system enables evaluations that are specifically dependent on d‐amino acids.

The optimized conditions for the inhibition assay were determined to be 0.2 U·mL^−1^ DAO, 2 mm d‐Ser, and 10% WST‐8. The calculated *K*_m_ value for 0.1–0.5 U·mL^−1^ DAO ranged from 3.151 (95% CI: 2.166–4.785) to 4.068 mm (95% CI: 2.278–8.29) (Table 1). Our use of 2 mm d‐Ser was deemed appropriate for these conditions, as Ooka et al. [13] reported that enzyme activity is maximal when the substrate concentration equals the *K*_m_ value. Additionally, in the cell viability assay using WST‐8, the manufacturer [22] indicates that absorbance values of < 3 are appropriate. Moreover, given that it is generally necessary for the enzymatic reaction to remain unsaturated, DAO at 0.2 U·mL^−1^ was not saturated at the 60‐min time point, whereas DAO at 1.0 U·mL^−1^ was already saturated by 20 min (Fig. S2). Therefore, our selected conditions are suitable because the reaction does not saturate within 60 min and the absorbance values fall within the appropriate analytical range. Furthermore, we confirmed that the selected pH allowed sufficient DAO activity without compromising the stability of the WST‐8 reagent. Finally, our method does not use Amplex Red, which requires a stop solution [9] (Table S2).

Subsequently, the inhibitory activity of two DAO inhibitors, SB and DAO‐IN‐2, was evaluated using this assay system. The resulting IC_50_ values were approximately 10 μm for SB [24] and < 1 μm for DAO‐IN‐2 [25]. These concentrations are within the range typically used for *in vitro* experiments. Furthermore, both compounds have been investigated for their potential applications as DAO inhibitors in animal studies and human clinical settings [8, 25, 26]. Therefore, the present assay system may serve as a practical *in vitro* screening platform for identifying compounds with potential *in vivo* applicability using known inhibitors as positive controls for comparison.

Although several DAO inhibitors have been reported, these compounds are small molecules, and considering that their inhibition mechanism follows a competitive inhibition pattern, it is highly plausible that they resemble amino acid metabolites [9, 19, 25]. Our investigation of the mode of inhibition revealed a competitive inhibition pattern (Fig. S7) for all molecules, except SB, which exhibited different inhibitory patterns at higher concentrations. This can be attributed to the biphasic inhibitory nature of SB [23, 27]. Uremic toxins are derived from dietary amino acids and often possess structures that include aromatic heterocycles conjugated with sulfate or acetate groups. These compounds share structural similarities with DAO inhibitors. Uremic toxins are generally excreted through the kidneys; however, they accumulate in the body when kidney function is impaired, such as in CKD, leading to a reduction in renal excretion [16]. These facts suggest that the relationship between uremic toxins and d‐amino acids is accompanied by impaired kidney function; however, the detailed mechanisms and causal relationships remain unclear. Nonetheless, this observation suggests a working hypothesis that uremic toxins inhibit DAO, thereby suppressing the metabolism of d‐amino acids and contributing to their accumulation in patients with CKD. In this study, we demonstrated that uremic toxins have endogenous inhibitory activity; however, their inhibitory effect was weaker than that of specific inhibitors. Notably, this study only evaluated previously reported uremic toxins; therefore, other unknown endogenous substances may have contributed to this inhibition. Additionally, previous studies using mouse models of acute kidney injury have reported that the blood concentration of d‐Ser increases, whereas the urinary concentration of d‐Ser decreases [28]. The mechanism underlying this phenomenon may involve a decrease in DAO expression and activity in the kidneys owing to ischemic acute kidney injury. Although information regarding DAO expression and activity in patients with CKD is lacking, damage to renal tubular cells is associated with decreased DAO expression levels [29]. Therefore, further investigations are needed to understand the details of d‐amino acid accumulation in chronic kidney disease.

This study had some limitations. First, the reaction system depends on redox reactions; thus, it cannot be used as a screening system for compounds that mediate redox activity. Additionally, all reducing compounds with SH groups, such as d‐Cys, l‐Cys, and N‐acetylcysteine, showed positive results. Although several evaluation systems for DAO and its inhibitory activity are available, some of these systems detect its activity using redox reaction mechanisms [9]. Therefore, it is important to consider this aspect, which can be applied to other reaction systems. Second, this reaction system has a lower sensitivity than those of fluorescence‐based assay systems. In this study, DAO activity was tested at concentrations of 100–200 mU·mL^−1^. Brugger et al. [10] tested DAO activity in the range of 500–5000 mU·mL^−1^ and found good sensitivity. A reaction system using Amplex Red® has shown that DAO activity can be evaluated in the range of 0.25–2.5 mU·mL^−1^; however, it is expensive compared with other detection systems [9] (Table S2). Additionally, the Amplex‐Red®‐based assay is more complex than our assay system (Table S2) as it requires a more expensive fluorescence detection instrument than a spectrophotometer, involves additional reagents and procedures owing to the use of coupling reactions with HRP, and requires a reaction stop reagent. Our method is simpler and more cost‐effective in all of these aspects. However, similar to other redox‐based systems, caution is required when screening compounds with redox activity. Third, we did not evaluate whether uremic toxins exhibited inhibitory activity *in vivo*. It is necessary to assess the changes in blood d‐amino acid levels upon administration in animals to clarify physiological activity in CKD. Fourth, in the present reaction system, we were unable to determine the precise electron transfer mechanism. Specifically, it is not clear whether the reduction of WST‐8 occurs via 1‐methoxy PMS contained in the Cell Counting Kit‐8 reagent or whether electrons generated by DAO directly reduce WST‐8 (Fig. 1). Due to patent‐related constraints, these mechanistic details could not be clarified.

In summary, we established another evaluation system for DAO‐inhibitory activity using the one‐step reagent WST‐8 and absorbance measurements using a plate reader. Furthermore, we demonstrate that endogenous uremic toxins inhibit DAO.

## Conflict of interest

The authors declare no conflict of interest.

## Author contributions

KM, YE, and YN were responsible for conceptualization, data curation, formal analysis, investigation, supervision, visualization, resource acquisition, and writing the original draft of the manuscript. YE was responsible for the methodology. KM, YE, YN, KT, and KM were responsible for project administration; and YE and KT were responsible for reviewing and editing the manuscript.

## Supporting information


**Table S1.** Liquid chromatography–tandem mass spectrometry conditions.
**Table S2.** Comparison of costs of DAO activity determination using the WST‐8 assay and horseradish peroxidase/Amplex UltraRed coupled assay.
**Fig. S1.** Evaluation of the effect of pH on DAO activity.
**Fig. S2.** Dose and time dependency of d‐amino acid oxidase.
**Fig. S3.** Quantification of d‐Ser after a 60‐min reaction using LC–MS/MS.
**Fig. S4.** Reactivity of other types of SH‐group compounds (N‐acetylcysteine or l‐cysteine [l‐Cys]) using the DAO detection assay.
**Fig. S5.** Evaluation of temperature as a DAO reaction stop condition.
**Fig. S6.** Validation assay.
**Fig. S7.** Double reciprocal plot analysis of DAO inhibitor and uremic toxins.

## Data Availability

All data supporting the findings of this study are available in the paper and Supporting Information.

## References

[1] Pollegioni L , Piubelli L , Sacchi S , Pilone MS and Molla G (2007) Physiological functions of D‐amino acid oxidases: from yeast to humans. Cell Mol Life Sci 64, 1373–1394. 17396222 10.1007/s00018-007-6558-4 PMC11136250

[2] Ross CA , Margolis RL , Reading SAJ , Pletnikov M and Coyle JT (2006) Neurobiology of schizophrenia. Neuron 52, 139–153. 17015232 10.1016/j.neuron.2006.09.015

[3] Mitchell J , Paul P , Chen HJ , Morris A , Payling M , Falchi M , Habgood J , Panoutsou S , Winkler S , Tisato V *et al*. (2010) Familial amyotrophic lateral sclerosis is associated with a mutation in D‐amino acid oxidase. Proc Natl Acad Sci USA 107, 7556–7561. 20368421 10.1073/pnas.0914128107 PMC2867752

[4] Pollegioni L and Sacchi S (2010) Metabolism of the neuromodulator D‐serine. Cell Mol Life Sci 67, 2387–2404. 20195697 10.1007/s00018-010-0307-9 PMC11115609

[5] Zhou Q and Sheng M (2013) NMDA receptors in nervous system diseases. Neuropharmacology 74, 69–75. 23583930 10.1016/j.neuropharm.2013.03.030

[6] Wu SZ , Bodles AM , Porter MM , Griffin WST , Basile AS and Barger SW (2007) Induction of serine racemase expression and D‐serine release from microglia by secreted amyloid precursor protein (sAPP). Curr Alzheimer Res 4, 243–251. 17627481 10.2174/156720507781077241

[7] Sacchi S , Rosini E , Pollegioni L and Molla G (2013) D‐amino acid oxidase inhibitors as a novel class of drugs for schizophrenia therapy. Curr Pharm Des 19, 2499–2511. 23116391 10.2174/1381612811319140002

[8] Lin CH , Lin CH , Chang YC , Huang YJ , Chen PW , Yang HT and Lane HY (2018) Sodium benzoate, a D‐amino acid oxidase inhibitor, added to clozapine for the treatment of schizophrenia: a randomized, double‐blind, placebo‐controlled trial. Biol Psychiatry 84, 422–432. 29397899 10.1016/j.biopsych.2017.12.006

[9] Rosini E , Caldinelli L and Piubelli L (2018) Assays of D‐amino acid oxidase activity. Front Mol Biosci 4, 102. 29404340 10.3389/fmolb.2017.00102 PMC5785730

[10] Brugger D , Krondorfer I , Zahma K , Stoisser T , Bolivar JM , Nidetzky B , Peterbauer CK and Haltrich D (2014) Convenient microtiter plate‐based, oxygen‐independent activity assays for flavin‐dependent oxidoreductases based on different redox dyes. Biotechnol J 9, 474–482. 24376171 10.1002/biot.201300336 PMC4162990

[11] Sacchi S , Cappelletti P , Giovannardi S and Pollegioni L (2011) Evidence for the interaction of D‐amino acid oxidase with pLG72 in a glial cell line. Mol Cell Neurosci 48, 20–28. 21679769 10.1016/j.mcn.2011.06.001

[12] Hopkins SC , Heffernan MLR , Saraswat LD , Bowen CA , Melnick L , Hardy LW , Orsini MA , Allen MS , Koch P , Spear KL *et al*. (2013) Structural, kinetic, and pharmacodynamic mechanisms of D‐amino acid oxidase inhibition by small molecules. J Med Chem 56, 3710–3724. 23631755 10.1021/jm4002583

[13] Ooka H , Chiba Y and Nakamura R (2023) Thermodynamic principle to enhance enzymatic activity using the substrate affinity. Nat Commun 14, 4860. 37620340 10.1038/s41467-023-40471-y PMC10449852

[14] Ishiyama M , Miyazono Y , Sasamoto K , Ohkura Y and Ueno K (1997) A highly water‐soluble disulfonated tetrazolium salt as a chromogenic indicator for NADH as well as cell viability. Talanta 44, 1299–1305. 18966866 10.1016/s0039-9140(97)00017-9

[15] Tominaga H , Ishiyama M , Ohseto F , Sasamoto K , Hamamoto T , Suzuki K and Watanabe M (1999) A water‐soluble tetrazolium salt useful for colorimetric cell viability assay. Anal Commun 36, 47–50.

[16] Duranton F , Cohen G , De Smet R , Rodriguez M , Jankowski J , Vanholder R and Argiles A (2012) Normal and pathologic concentrations of uremic toxins. J Am Soc Nephrol 23, 1258–1270. 22626821 10.1681/ASN.2011121175 PMC3380651

[17] Vanholder R , Schepers E , Pletinck A , Nagler EV and Glorieux G (2014) The uremic toxicity of indoxyl sulfate and p‐cresyl sulfate: a systematic review. J Am Soc Nephrol 25, 1897–1907. 24812165 10.1681/ASN.2013101062 PMC4147984

[18] Gryp T , Vanholder R , Vaneechoutte M and Glorieux G (2017) p‐cresyl sulfate. Toxins 9, 52. 28146081 10.3390/toxins9020052 PMC5331431

[19] Smith SM , Uslaner JM and Hutson PH (2010) The therapeutic potential of D‐amino acid oxidase (DAAO) inhibitors. Open Med Chem J 4, 3–9. 20648222 10.2174/1874104501004020003 PMC2905773

[20] Harris CM , Molla G , Pilone MS and Pollegioni L (1999) Studies on the reaction mechanism of Rhodotorula gracilis D‐amino‐acid oxidase. Role of the highly conserved Tyr‐223 on substrate binding and catalysis. J Biol Chem 274, 36233–36240. 10593911 10.1074/jbc.274.51.36233

[21] Nakano Y , Konya Y , Taniguchi M and Fukusaki E (2017) Development of a liquid chromatography‐tandem mass spectrometry method for quantitative analysis of trace D‐amino acids. J Biosci Bioeng 123, 134–138. 27542694 10.1016/j.jbiosc.2016.07.008

[22] Cell counting kit‐8 CK04 manual DOJINDO. https://www.dojindo.co.jp/products/CK04/

[23] Murtas G , Sacchi S , Valentino M and Pollegioni L (2017) Biochemical properties of human D‐amino acid oxidase. Front Mol Biosci 4, 88. 29326945 10.3389/fmolb.2017.00088 PMC5737116

[24] Toguchi S , Hirose T , Yorita K , Fukui K , Sharpless KB , Omura S and Sunazuka T (2016) In situ click chemistry for the identification of a potent D‐amino acid oxidase inhibitor. Chem Pharm Bull 64, 695–703. 10.1248/cpb.c15-00867 26686243

[25] Sparey T , Abeywickrema P , Almond S , Brandon N , Byrne N , Campbell A , Hutson PH , Jacobson M , Jones B , Munshi S *et al*. (2008) The discovery of fused pyrrole carboxylic acids as novel, potent D‐amino acid oxidase (DAO) inhibitors. Bioorg Med Chem Lett 18, 3386–3391. 18455394 10.1016/j.bmcl.2008.04.020

[26] Modi KK , Roy A , Brahmachari S , Rangasamy SB and Pahan K (2015) Cinnamon and its metabolite sodium benzoate attenuate the activation of p21rac and protect memory and learning in an animal model of alzheimer's disease. PLoS One 10, e0130398. 26102198 10.1371/journal.pone.0130398 PMC4478015

[27] Caldinelli L , Molla G , Sacchi S , Pilone MS and Pollegioni L (2009) Relevance of weak flavin binding in human D‐amino acid oxidase. Protein Sci 18, 801–810. 19309736 10.1002/pro.86 PMC2762592

[28] Sasabe J , Suzuki M , Miyoshi Y , Tojo Y , Okamura C , Ito S , Konno R , Mita M , Hamase K and Aiso S (2014) Ischemic acute kidney injury perturbs homeostasis of serine enantiomers in the body fluid in mice: early detection of renal dysfunction using the ratio of serine enantiomers. PLoS One 9, e86504. 24489731 10.1371/journal.pone.0086504 PMC3906037

[29] Zhang H , Qi L , Lin Y , Mao L and Chen Y (2012) Study on the decrease of renal D‐amino acid oxidase activity in the rat after renal ischemia by chiral ligand exchange capillary electrophoresis. Amino Acids 42, 337–345. 21110210 10.1007/s00726-010-0811-0

