# Supplementary material for: Establishing an assay to evaluate d‐amino acid oxidase enzyme kinetics and inhibition using WST‐8 redox dye
Source: FEBS Open Bio. 2026 Feb 24;16(9):1645–56. doi: 10.1002/2211-5463.70217 (PMC13398905; doi:10.1002/2211-5463.70217)
Supplement: Supplementary file 1 [file FEB4-16-1645-s001.docx]

**Supplementary Information**

**Supplementary Table S1 LC-MS/MS conditions**

| **Instrument** | |
| --- | --- |
| LC system | Prominence-i LC-2023C (Shimadzu) |
| Valve Unit | FCV-20AH2 (Shimadzu) |
| MS system | LCMS-8050 (Shimadzu) |
|  | |
| **Analytical conditions** | |
| Analytical column | Daicel, Crownpak CR-I (+), 150×3 mm, 5 μm |
| Guard column | YMC-Guardpack Diol-300, 30×8.0mml.D.S-5 μm, 30 nm |
| Column oven temp. | 30°C |
| Flow rate | 0.3 mL/min |
| Injection vol. | 5 μL |
| Mobile phase | AcCN:EtOH:ultra-pure water: TFA = 95:3:2:0.5 |
| Gradient condition | None. |
| Measurement time | 8 min |
| Ion mode | MRM (positive ion mode) |
| Interface voltage | 4 kV |
| CID gas | 230 kPa |
| Interface temp. | 300°C |
| DL temp. | 250°C |
| Heat block temp. | 400°C |
| Nebulizer gas flow rate | 3 L/min |
| Heating gas flow rate | 10 L/min |
| Drying gas flow rate | 10 L/min |

*AcCN*, acetonitrile; *EtOH*, ethanol; *TFA*, trifluoroacetic acid;, *MRM* multiple reaction monitoring.

**Supplementary Table S2. Comparison of DAO activity determination assay cost between WST-8 assay and horseradish peroxidase/Amplex UltraRed coupled assay**

| Contents | WST-8 assay | Amplex UltraRed coupled assay | Cost* |
| --- | --- | --- | --- |
| Equipment | Microplate reader: Absorbance (450 nm) | Microplate reader: Fluorescent (Ex/Em = 571/581nm) | $$$ |
| Plate | General 96 well microplate (clear) | General 96 well black microplate (black) | $$ |
| Buffer | 50 mM sodium phosphate buffer | 50 mM sodium phosphate buffer | $ |
| Reagents/test | - Detection: WST-8 - Procine DAO - D-serine   Reaction scale: 100 µL | - Detection: Amplex Ultrared - Human DAO - D-serine - FAD - HRP   Reaction scale: 200 µL | $$$ |
| Procedure | *Identification of DAO inhibitors*   1. The mixtures are prepared in a 96 wellplate by mixing 10 µL of 2 U/mL DAO and 10 µL of WST-8 with 10 µL of diﬀerent inhibitors, in 50 mM sodium phosphate buﬀer, pH 8.0. 2. Subsequently, 10 µL of D-serine is added and absorbance is measured at 0 min (450 nm). 3. After 60 min, absorbance is measured again (450 nm). | *Identification of DAO inhibitors***   1. The preincubation mixtures are prepared in Eppendorf-type test tubes by mixing 25 µL of 0.01 mg/mL DAO with 625 µL of diﬀerent inhibitors, in 50 mM sodium phosphate buﬀer, pH 7.4, 4 µM FAD. 2. The tubes are incubated for 30 min at room temperature (25°C). 3. Preparation of the reaction mixture: 130 µL of preincubation mixture is transferred to each well of a microplate (four wells for each concentration of the inhibitor is tested) and 70 µL of WSC (22 mM D-serine, 4 µM FAD, 0.1 mM Amplex R UltraRed Reagent, 0.2 U/mL HRP, in 50 mM sodium phosphate buﬀer, pH 7.4) are added. 4. The reaction is stopped by adding 40 µL of Stop Reagent solution after 30 min. 5. The ﬂuorescence value determined for the control in the absence of the compound is used as reference (activity =100%). |  |
| Total |  |  | $$$ |

*Cost: The cost is presented as the relative price with the WST set to 1 (=$).

Ex/Em: excitation/emission

Porcine DAO was purchased from Merck Co., Ltd.

Human recombinant DAO is produced in *Escherichia coli*

*FAD*, flavin adenine dinucleotide; *HRP* horseradish peroxidase.

**Supplementary Figure S1. Evaluation of the effect of pH on DAO activity**

(A) Verification of the pH of the phosphate buffer used in the experiments after adjustment. (B, C) Appearance of each well after reacting d-Serine (d-ser) (2 mM) with d-amino acid oxidase (DAO) (0.2 U/mL) at 37°C for 60 min under different pH conditions.

**Supplementary Figure S2.** **Dose and time dependency of d-amino acid oxidase**

d-Amino acid oxidase (DAO) concentration ranges from 0.01–1.0 U/mL; d-Serine (d-Ser) concentration ranges from 0.1–10 mM. The temperature was set at 37°C, and measurements were taken every 10 min. Data are presented as the mean of n = 2.

**Supplementary Figure S3. Quantification of d-Ser after a 60-min reaction using LC-MS/MS**

After reacting d-Serine (d-Ser) (2 mM) at 37°C for 60 min in the presence or absence of d-amino acid oxidase (DAO) (0.2 U/mL), the residual d-Ser in the reaction mixture was measured by LC–LC-MS/MS. Data are presented as the mean of n = 2.

**Supplementary Figure S4. Reactivity of other types of SH-group compounds (N-acetylcysteine or l-cysteine [l-Cys]) using the DAO detection assay**

d-Amino acid oxidase (DAO) (0.2 U/mL) and each substrate (2 mM) were added, and the change in absorbance was evaluated after reacting at 37°C for 60 min. Data are presented as the mean of n = 2.

**Supplementary Figure S5. Evaluation of temperature as a DAO reaction stop condition.**

Under conditions of 2 mM D-Serine (D-ser) and 0.1 or 0.2 U/mL D-amino acid oxidase (DAO), the change in absorbance after 60 min was evaluated at temperatures of 4°C or 37°C. Data are expressed as means ± SE. n = 3 per experiment.

**Supplementary Figure S6. Validation assay**

The validation assay was performed by the following conditions: 0.2 U/mL d-amino acid oxidase (DAO), d-Ser (final conc. 0.1–10 mM) at temperature of 37°C in phosphate buffer (pH 8.0). Data are expressed as means ± SE. n = 3 per experiment.

**Supplementary Figure S7. Double reciprocal plot analysis of DAO inhibitor and uremic toxins**

Sodium benzoate [SB], indoxyl sulfate [IS], indoxyl acetic acid [IA], p-cresol sulfate [PCS], kynurenic acid [KA], and hippuric acid [HA]. Data are presented as the mean of n = 2.
